# What Then?

**Published:** 1914-08

**Authors:** 


### What Then?

Granting that sexual instinct is a natural function of the body, subject to the operation of physical laws, the same as any other function;

That it knows no moral or unmoral, marital or extra marital relations such as is prescribed by social ethics;

That its normal exercise is essential to the development of a strong physical and mental body;

That its repression is harmful to the physical and mental welfare of the individual, often being the causative factor in many intractable mental and nervous diseases;

That it is the msot impelling force of the human race for the gratification of which, under the most favorable condition, society was founded and is continued;

That marriage is only a civil contract for the better protection of the offspring and property rights;

That marriage does not constitute the moral right which is founded on mutual consent and personal interest: Granting all this to be true from a strictly physiological standpoint, *what then?*

What then! You have considered but one *phase* of the question. You have only dealt with the *materialistic* facts. You have not considered the higher ethical principles—the *moral mores* which has directed cultured progress since the dawn of civilization. Like physical character the *mores* has its specific influence on human development and in determining what shall be the social and ethical standard of the race or nation. To alter their influence would affect the social code of the classes as surely as artificial selection would change the physical characteristics.

Consider what would be the result if an organic change were effected in our social mores. Suppose society no longer reverenced the honor of woman or stood ready to protect her virtues; that constancy and devotion no longer remained as the reward of faithful love; that the fireside no longer shielded those sacred rights of plighted love that a man of honor would not seek to cross? Suppose this condition changed, what would be the status of society? Virtue would cease to have a value! There would be no incentive to personal sacrifice and striving for a coveted goal! Woman would no longer be the high priestess of love and honor! She would be valued only for her material worth and readiness to serve a physical demand. There would be no need of marriage save for legal purposes. The home, the "unite of society," would exist for convenience solely and for economic purposes. There would be little for organized society to protect; brute force would rule where justice once reigned. *What then?*

The writer's apology for this pronouncement, if any be needed, is because of the growing notion, advanced by the so-called radicals of the eugenics school, who would reduce everything socially and ethically to the material basis of sex, who hold that "life is one long sex pursuit." A broader knowledge of sex and its physical relations is sadly needed, and, while its practice would add materially to our happiness and to a saner way of living, life, stripped of its ethical side, would be like the brute creation and without *human* interest.

